# Does NLRP3 Inflammasome and Aryl Hydrocarbon Receptor Play an Interlinked Role in Bowel Inflammation and Colitis-Associated Colorectal Cancer?

**DOI:** 10.3390/molecules25102427

**Published:** 2020-05-22

**Authors:** Ivan Qi Han Ngui, Agampodi Promoda Perera, Rajaraman Eri

**Affiliations:** School of Health Sciences, University of Tasmania, Launceston, TAS 7248, Australia; iqhngui@utas.edu.au (I.Q.H.N.); agampodi.perera@utas.edu.au (A.P.P.)

**Keywords:** aryl hydrocarbon receptor, NLRP3 inflammasome, colitis, colon cancer, NF-κB

## Abstract

Inflammation is a hallmark in many forms of cancer; with colitis-associated colorectal cancer (CAC) being a progressive intestinal inflammation due to inflammatory bowel disease (IBD). While this is an exemplification of the negatives of inflammation, it is just as crucial to have some degree of the inflammatory process to maintain a healthy immune system. A pivotal component in the maintenance of such intestinal homeostasis is the innate immunity component, inflammasomes. Inflammasomes are large, cytosolic protein complexes formed following stimulation of microbial and stress signals that lead to the expression of pro-inflammatory cytokines. The NOD-, LRR- and pyrin domain-containing protein 3 (NLRP3) inflammasome has been extensively studied in part due to its strong association with colitis and CAC. The aryl hydrocarbon receptor (AhR) has recently been acknowledged for its connection to the immune system aside from its role as an environmental sensor. AhR has been described to play a role in the inhibition of the NLRP3 inflammasome activation pathway. This review will summarise the signalling pathways of both the NLRP3 inflammasome and AhR; as well as new-found links between these two signalling pathways in intestinal immunity and some potential therapeutic agents that have been found to take advantage of this link in the treatment of colitis and CAC.

## 1. Introduction

Inflammatory bowel disease (IBD) is a chronic inflammatory disorder of the gut that includes ulcerative colitis (UC) and Crohn’s disease (CD) [1]. While the incidence of IBD only occurs in 1–2% of all colorectal cancer (CRC) patients in the general population [2], this incidence has been quantified to increase up to 5% in patients suffering over 20 years of the disease [3]. The risk of CRC development rises in patients with IBD and is a more belligerent carcinoma with an earlier age of onset than sporadic CRC [2]; with the major driving force of carcinogenesis being inflammation and immunosuppression [4]. This has led to a poor prognosis for patients with IBD, with 1 in 6 patients dying from cancer [5]. Molecular changes and mutations in colitis-associated colorectal cancer (CAC) are linked to inflammation-driven by dysregulated cytokines and inflammatory mediators corresponding to IBD, which alter cell communication, cell-to-cell signalling and cell adhesion [6]. In the Australian context, colorectal cancer is projected to become the most expensive cancer to treat; with financial costs of treatment estimated to increase from AUS$1 billion in 2013 [7] to AUS$2 billion in 2040, excluding screening programs [8]. Recently, inflammasomes have been recognised as the main driving force in the mucosal inflammatory process.

Inflammasomes are large cytosolic protein complexes that have been recognised as a major component of the innate immunity [9]. These high molecular weight complexes function to mediate the host defence mechanism against microbial infections and danger-associated endogenous stimuli [9,10] The initiation of inflammatory signalling cascades that lead to the proteolytic cleavage of pro-caspase-1 into caspase-1 that activates the cytokine precursors, pro-IL-1β and pro-IL-18, leading to pyroptosis [11,12]. Pattern-recognition receptors (PRRs), which consist of toll-like receptors (TLRs) and nod-like receptors (NLRs), serve as the initiators of this signalling cascade when they recognise pathogen-associated molecular patterns (PAMPs) and danger-associated molecular patterns (DAMPs), resulting from pathogens, endogenous stress and particulate matters [13]. 

In this review, we will comprehensively describe the role that the aryl hydrocarbon receptor (AhR) plays in the inhibition of NLRP3 inflammasome and its effects on CRC. While some studies have described the strong association between AhR and NLRP3, they have not explored the cross-over between the signalling pathways of AhR and NLRP3 extensively. The signalling pathways of AhR will be described and the link between these pathways with the NLRP3 signalling pathways will be explored. The therapeutic potential of AhR will also be discussed, with examples of novel therapeutic agents that involve the immunomodulation of AhR.

## 2. Biological Features of the NLRP3 Inflammasome

The NLRP3 inflammasome is one of the most extensively studied NLRs due to its clinical relevance in a wide range of human diseases. This 115 kDa cytosolic protein complex consists of a triadic constitution; the NACHT scaffold which serves as a central oligomerization domain with an ATPase activity, the N-terminal PYCARD adaptor which recruits the apoptosis-associated speck-like protein (ASC) and the C-terminal leucine-rich repeat (LRR) which are thought to be involved in detecting stimuli [11,14]. 

### 2.1. The NLRP3 Inflammasome Canonical Pathway

**Figure 1:**
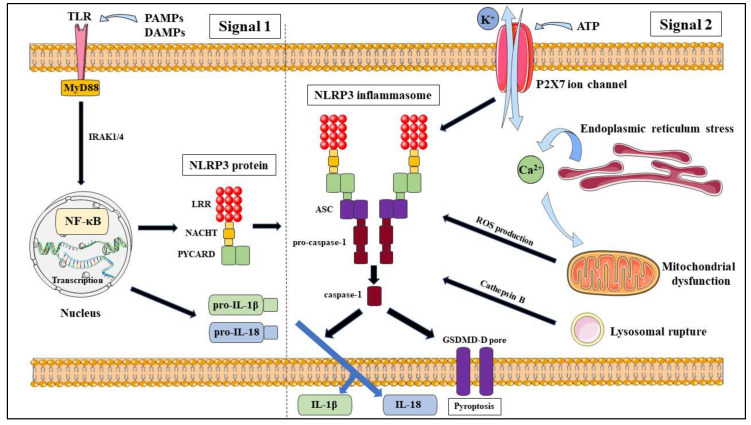
Canonical activation pathway of nod-like receptor (NLR)P3 inflammasome.

The canonical pathway (Figure 1) requires a two-step process which is proposed to be mediated at both the transcriptional and post-transcriptional levels [15], known as priming (signal 1) and activation (signal 2). Signal 1 involves a priming signal that is generated by TLR4 agonists through the TLR/nuclear factor (NF)-kB pathway that positively induces the expression of NLRP3, pro-IL-1β and pro-IL-18. Signal 2 involves the detection of PAMPs and DAMPs that lead to the formation of the NLRP3 inflammasome [15,16].

Priming is a necessary step in canonical NLRP3 activation as it ensures that NLRP3 activation is a controlled process to prevent harmful effects on the host and that once priming commenced, the macrophage would commit to activating the NLRP3 inflammasome [17]. Within the priming step itself, there are also several pathways identified; which can be classified as transcriptional and non-transcriptional pathways [18]. The transcriptional priming pathway (>3 h) involves the activation of the NF-κB signalling pathway; with Bauernfeind et al. concluding that the disruption to the NF-κB signalling pathway activators or its components; such as MyD88, IRAK1 and IRAK4, lead to the reduction in NLRP3 expression in human monocytes, even when treated with signal 2 [17]. The non-transcriptional pathways both rely on post-transcriptional modifications (PTM) and mitochondrial ROS and can be effective within 10–30 min (MyD88 binding) or 30–60 min (TRIF binding) after TLR activation through their own respective pathways [18]. Through PTM by phosphorylation and ubiquitination, the lengthy duration for transcriptional priming can be bypassed, providing rapid priming of NLRP3. The PTM of NLRP3 inflammasome during priming will be discussed further below. 

Following priming, NLRP3 is licenced to be activated by its diverse activation signals; but not directly bound to the complex [11], that leads to the secretion of the proinflammatory cytokines IL-1β and IL18; as well as stimulate pyroptosis in the cell through disrupting the osmotic potential due to cytoplasmic Gasdermin-D (GSDMD-D) forming pores [18]. There would be an underlying molecular mechanism to allow these activation signals; PAMPs and DAMPs, to indirectly activate the NLRP3 inflammasome; which have been proposed to be through ion fluxes, mitochondrial dysfunction and ROS production, cathepsin B and lysosomal rupture and PTM of NLRP3 [19].

#### 2.1.1. Ion Fluxes

The efflux of K^+^ is an established model that is an important component to the activation of NLRP3 inflammasome, which precedes mitochondrial dysfunctions [20] and proposed to occur upstream of NLRP3 activation, further supporting that K^+^ efflux takes place prior to NLRP3 activation [11]. The generic NLRP3 activators, ATP and nigericin, modify intracellular components and decrease intracellular K^+^ concentrations through non-selective K^+^ conduction out of the cell across the plasma membrane [15] by pores formed on the plasma membrane; such as the P2X7 ATP gated ion channels [19], which lead to a conformational change in NLRP3 inflammasome-forming oligomers [18]. While K^+^ is important, it is unspecific and not required for NLRP3 inflammasome activation as there are studies that have shown that small molecules; such as imiquimod and CL097 [21], and macrophage models that have the NLRP3 activating mutation (NLRP3^R258W^) can still activate NLRP3 independent of K^+^ efflux [11]. There is also evidence that supports the notion that Cl^−^ efflux can also lead to the activation of NLRP3; through the use of NSAIDs that inhibit the membrane Cl^−^-channel, VRAC, which suppressed NLRP3 activation, by interfering with NLRP3-NEK7 interactions [16,22,23]. While there is evidence of Na^+^ influx and Zn^2+^ inducing NLRP3 activation, they are not conclusive and there is a possibility that Na^+^ influx is dependent on K^+^ efflux [16,21]. Ca^2+^ signalling has also been associated with NLRP3 inflammasome activation. There are propositions to how an increased Ca^2+^ is integrated with NLRP3 activation, which describe that a rise in extracellular K^+^ promotes both K^+^ efflux and an increased cytosolic Ca^2+^, inferring a link between K^+^ and Ca^2+^ ionic balance [18] or that Ca^2+^ overload due to excessive release from storage in the endoplasmic reticulum leads to mitochondrial dysfunction, which results in the production of ROS [16,21]. However, further studies are required to determine its significance and understand the mechanisms behind Ca^2+^ signalling in the activation of NLRP3 inflammasome. 

#### 2.1.2. Mitochondrial Dysfunction and ROS Production

Another proposed model of NLRP3 inflammasome activation is the production of cytosolic ROS by NADPH oxidase following mitochondrial dysfunction in consequence of endogenous stress from the detection of various NLRP3 agonists [11,16,19]. The influence of mitochondrial dysfunction on NLRP3 activation was identified from several studies that manipulated mitochondrial ROS production through induction with agonists and antagonists. This was achieved through the inhibition of NLRP3 activation via the blocking of mitochondrial ROS production [24]. Contrary to this, another study found that the stimulation of mitochondrial ROS production promoted NLRP3 activation [25]. Wen et al. demonstrated that inhibition of autophagy to remove non-functioning mitochondria via AMPK pathway, lead to upregulated ROS production and NLRP3 activation and IL-1β release, in response to LPS [26]. Following the detection of NLRP3 agonists, thioredoxin-interacting protein (TXNIP) activates the NLRP3 inflammasome via a ROS-dependent manner through binding to the LRR domain of NLRP3 [19]. The study further demonstrated that there was a deficiency in the activation of caspase-1 and expression of IL-1β during inhibition of TXNIP; which suggests that there are other models that work in tandem with the ROS model to regulate NLRP3 activation [19]. Another study also implicated the role of mitochondrial antiviral-signalling protein (MAVS) in NLRP3 activation but was unclear on the mechanism regulating its role [27]. Therefore, further studies must be conducted to determine the role that ROS model plays in NLRP3 activation. 

#### 2.1.3. Cathepsin B and Lysosomal Rupture

In addition to ion fluxes and ROS production, lysosomal rupture has also been proposed as a model for NLRP3 inflammasome activation. Lysosomal rupture occurs from endocytosis of particulate matter, microbial pathogens and sterile environmental matter; which results in the release of the lysosomal protein cathepsin B [15]. Even though cathepsin B is the primary focus, other cathepsins have been indicated to also promote IL-1β secretion and NLRP3 inflammasome activation [28]. This is the case in a study by Duewell et al. which exhibited a reduction in IL-1β secretion when induced by cholesterol crystals to activate the NLRP3 inflammasome in mouse models deficient in cathepsin B or L as compared to wild types, and that phagolysosomal rupture was an essential event in NLRP3 activation [29]. Another study determined that the irreversible cathepsin B inhibitor, CA-074Me, inhibited NLRP1b-mediated caspase-1 activation when triggered by anthrax lethal toxins [30]. There is also evidence that demonstrated that Group B streptococci, malarial parasites and adenovirus type-5 require lysosomal rupture induce NLRP3 inflammasome activation [31,32,33]. There is also a link established between lysosomal rupture and K^+^ efflux, similar to ROS production; further solidifying the notion of K^+^ efflux being the common trigger for NLRP3 inflammasome activation. Another recent study by Munoz-Planillo et al. found that LPS priming stimulated an increased K^+^ efflux when activated by particulate matter; including the lysosomal-damaging dipeptide LL-OMe, due to the K^+^ efflux opening one or multiple K^+^ permeable membrane pores [20].

### 2.2. The NLRP3 Inflammasome Non-Canonical Pathway

**Figure 2:**
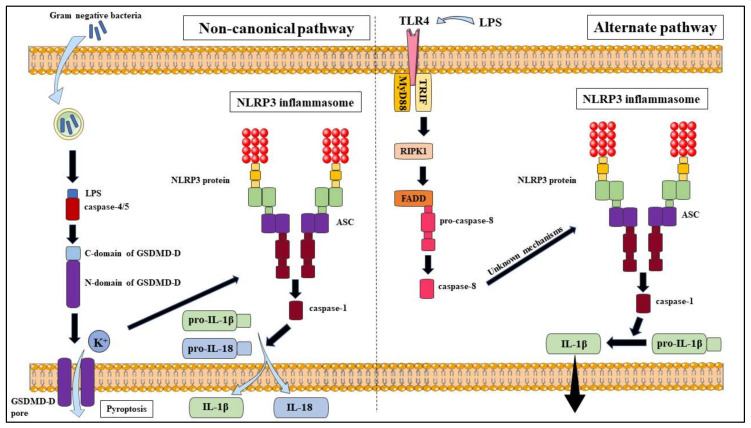
Non-canonical and alternate activation pathways of NLRP3 inflammasome.

The non-canonical pathway (Figure 2) is another NLRP3 activation pathway model that differs slightly from the canonical pathway due to the utilisation of caspase-4/5 (in humans) or caspase-11 (in mice) to release the proinflammatory cytokines, IL-1β and IL-18 and triggering pyroptosis. This pathway can be activated by most Gram-negative bacteria but not Gram-positive bacteria, which suggests that LPS plays a major role in this pathway [11,16]. Priming is a necessary step in the non-canonical pathway in mouse models due to reduced caspase-11 expression in resting cells; however, this is not the case in human models due to the expression of caspase-4 in many non-monocytic cells and monocytes [16]. Intracellular LPS is able to bind directly to the CARD domain of caspase-4/5/11 to stimulate oligomerisation and activation of these caspases, which cleave GSDMD-D into two fragments; N-domain and C-domain, that lead to the formation of pores on the plasma membrane and allow K^+^ efflux and pyroptosis to occur [11,16]. However, the caspases will not cleave and activate pro-IL-1β and pro-IL-18; instead, K^+^ efflux due to the pores formed will induce NLRP3 activation through the canonical pathway, inferring a link between the canonical and non-canonical pathway [16]. 

### 2.3. The NLRP3 Inflammasome Alternate Pathway

While both the canonical and non-canonical pathways follow a two-signal mechanism, there is evidence to show that human monocytes; unlike macrophages, can be triggered by TLR ligands alone; such as LPS. This process is done in the absence of K^+^ efflux; the common NLRP3 activation model, to activate caspase-1 and release mature IL-1β through the ATP-P2X7 pathway, without causing cell death [34,35]. This separation between the release of proinflammatory cytokines and non-reversible pyroptosis diversifies the roles the host defence can play in a greater variety of situations. This pathway (Figure 2) requires the activation of caspase-8 by cleaving through the TLR4–TRIF–RIPK1–FADD signalling pathway; though the actual mechanisms to cleave caspase-8 is still unclear [36]. Antonopoulos et al. suggested that caspase-8 may substitute the role of caspase-1 in inducing pore formation during pyroptosis and IL-1β processing [37]. This is in line with the results observed by Chung et al. who also identified that caspase-8 forms a complex with NLRP3 and ASC to induce apoptosis [38]. This stance is supported by Gringhuis et al. who identified that the dectin-1 PRR elicited pro-IL-1β activation following the detection of a specific fungal ligand via a caspase-8 dependent mechanism [39]. Contrary to this notion, Gaidt et al. have proposed that caspase-8 does not directly cleave IL-1β due to failure for *Nlrp3*^-/-^, *Asc*^-/-^ and *Caspase-1*^-/-^ monocytes to secrete IL-1β after LPS induction; and that the NLRP3 inhibitor, MCC950 can also block activation of the alternate pathway [40]. Additional studies are required to clarify the specific mechanisms involved but it is clear that caspase-8 plays an integral role in the alternate pathway activation of the NLRP3 inflammasome.

### 2.4. Regulation of NLRP3 Inflammasome 

As previously mentioned, PTM occurs in both the priming and activation step of the NLRP3 inflammasome and each component is important to maintain balance in the inflammatory response [41]. 

#### 2.4.1. Post-Translational Modifications during the Priming Step

The priming step of NLRP3 inflammasome can be shortened by utilising a non-transcriptional mechanism through TLR4-MyD88 signalling [42,43]. This suggests that regulation of PTM during priming is essential as part of the NLRP3 inflammasome activation pathway, in addition to transcription. The ubiquitin system consists of the addition of ubiquitin; ubiquitination, and the removal of ubiquitin; deubiquitination, both of which are part of the PTM of protein during NLRP3 inflammasome activation. An important study outlined the role that PAMPs and DAMPs play in modulating deubiquitinase activity. This same study also identified USP7 and USP47 as integral components in canonical NLRP3 inflammasome activation [44], further highlighting the importance of deubiquitinases. The direct phosphorylation of NLRP3 at Ser194 (Ser198 in humans [18]) by c-Jun terminal kinase 1 (JNK1), which has interactions with BRCA1–BRCA2-containing complex subunit 3 (BRCC3, a Lys63-specific deubiquitinase), has been shown to play a central role in NLRP3 deubiquitination during NLRP3 priming [45,46,47]. Humpries et al. provided the first description of the role played by E3 ubiquitin ligase Pellino2 in mediating the K63-linked ubiquitination of the NLRP3 inflammasome priming; through ubiquitination of the negative regulator IRAK1 [48]. In addition to that, phosphorylation of IKKs also have a role in NLRP3 inflammasome priming; with IKKα phosphorylation at S193 and S16 of ASC allowing sequestration of ASC in the nucleus, IKKi phosphorylation at S58 facilitating ASC translocation from the nucleus to the cytoplasm [49] and IKKγ/NF-κB essential modulator (NEMO) phosphorylation at S337 suppressing NF-κB activation [50]. Aside from NF-κB being involved in PTM, several components of MAPK were also found to have an impact in the priming step of NLRP3 activation via PTM. The external signal regulated kinase1 (ERK1) has been implicated in the NLRP3 inflammasome priming event; with a study by Ghonime et al. employing Erk1 knockdown mouse models leading to impaired priming [51]. This notion was supported by D’Espessailles et al. which found that G protein-coupled calcium-sensing receptor (CaSR)-dependent NLRP3 inflammasome activation uses the ERK1/2 signalling pathway [52]. This was also the case for p38 MAPK phosphorylation being an essential step in NLRP3 inflammasome priming with several studies showing a reduction in p38 activation through inhibition studies. These studies found that p38 MAPK signalling inhibitor SB203580 reducing NLRP3 inflammasome expression [53]. They also determined that Levistilide A reducing NLRP3 inflammasome expression through blocking of the Syk-p38/JNK pathway [54]. Blocking of the NLRP3-associated stress signals; K^+^ efflux, ROS production and cathepsin B, was also concluded to lead to a blockade of p38δ MAPK activation [55]. A series of studies also establish that C5a-C5aR2 complement crosstalk with protein kinase R (PKR) promotes NLRP3 inflammasome activation via MEK/ERK signalling and type I IFN signalling [56,57,58]. Finally, there was also a study by Basak et al. which illustrated that LPS derived from H. pylori can lead to the phosphorylation of caspase-1 at Ser376 through the PI-3K/Rac1/p21-activated kinase (PAK) 1 signalling pathway and is crucial for activation of caspase-1 [59]. 

#### 2.4.2. Post-Translational Modifications During the Activation Step

In addition to PTM during the NLRP3 inflammasome priming step, a diverse range of PTM has also been described in its activation step. Protein kinase A (PKA) has been found to negatively regulate NLRP3 activation by phosphorylation at Ser295 (Ser291 in mouse models) in the NACHT domain; which is the site for cryo-pyrin-associated periodic syndromes (CAPS)-associated NLRP3 mutations [60], via TGR5-cAMP-PKA axis [61] and prostaglandin E2 receptor E-prostanoid 4 (EP4) axis [62]. In contrast to this, Zhang et al. found that Golgi-mediated protein kinase D (PKD) phosphorylation at the Ser295 site leads to the release of mitochondria-associated membrane (MAM) that promotes NLRP3 inflammasome activation [63]; further solidifying Ser295 phosphorylation as an important event in the regulation of the NLRP3 inflammasome activation. The E3 ligase, Ariadne homolog 2 (ARIH2) was also shown to negatively regulate NLRP3 inflammasome activity in macrophages via NLRP3 ubiquitination linked through K48 and K63 in the NACHT domain [64]. In addition to ARIH2, there are other E3 ligases that have implications in NLRP3 inflammasome activation; with Tripartite Motif Containing 31 (TRIM31) promoting K48-linked polyubiquitination and proteosomal degradation of NLRP3 [65] and F-box L2 (FBXL2) promoting ubiquitin ligation at Lys-689 within NLRP3 and promote its degradation [66]. Similar to FBXL2, F-box-only protein 3 (FBXO3); another F-box protein, is involved in the LPS-induced NLRP3 inflammasome priming and is responsible for the ubiquitination and degradation of FBXL2 [66]. There is also evidence that shows that the neurotransmitter, dopamine, negatively regulates NLRP3 inflammasome activation via the cAMP-mediated dopamine D1 receptor (DRD1) signalling pathway by promoting its ubiquitination and degradation via the E3 ubiquitin ligase, MARCH7 [67]. In addition to protein kinases, Stutz et al. have described phosphatases to be involved in the regulation of the NLRP3 inflammasome; such as phosphatase 2A (PP2A) dephosphorylating the pSer5 residue at the NLRP3 PYD domain to promote NLRP3 inflammasome activation [68]. In addition, Spalinger et al. described protein tyrosine phosphatase non-receptor 22 (PTPN22) to dephosphorylate the Tyr861 site to prevent excessive NLRP3 inflammasome activation [69]. 

### 2.5. The NLRP3 Inflammasome and IBD

There is still a disparity in the results from many studies that describe the role NLRP3 plays on colitis as a protective or detrimental one [12]. However, recent studies have shown the success of NLRP3 inhibitors in ameliorating colitis and in turn CAC, providing strong evidence that an overactivation of NRLP3 is detrimental to intestinal homeostasis. Some inhibitors act to directly disrupt functional components of the NLRP3 inflammasome activation pathway. MCC950 has been found to suppress the transcription and translation of IL-1β and IL-18 in both canonical and non-canonical pathways [70]. Curcumin was determined to prevent the key events in the activation step (Signal 2) of the NLRP3 activation pathway; which include K^+^ efflux, ROS formation and cathepsin B release, thus preventing NLRP3 inflammasome activation [71]. Wogonoside was described to inhibit NF-κB activity, which prevents NLRP3 activation in the colon [72]. On the other hand, other inhibitors have attenuated the effects of colitis through indirect interactions with the NLRP3 inflammasome. Arctigenin acts to inhibit NLRP3 inflammasome activation via SIRT1, which functions to suppress the expression of inflammatory genes [73]. Palmatine was found to facilitate a specialised autophagy pathway, the PINK1/Parkin-driven mitophagy, which inactivates NLRP3 in macrophages [74]. Formononetin was described to promote the expression of epithelial cell tight junctions, which are prominently reduced in DSS-induced colitis, and maintained the colonic epithelial barrier [75]. Cinnamaldehyde was described to ameliorate ulcerative colitis through the suppression of miR-21 and miR-155 in the colon and macrophage [76]. 

It is clear that IL-1β is an integral component of the pool of pro-inflammatory cytokines present during inflammation in IBD patients [77]. The role of the NLRP3 inflammasome as a regulator of IL-1β maturation strengthens the notion that it plays a vital role in the development of IBD. As previously mentioned, several studies utilised NLRP3 inhibitors to ameliorate the effects of DSS-induced colitis. A recent study investigated this premise through a different perspective; by utilising an NLRP3 activator, the Jumonji domain-containing 3 (Jmjd3). This study found that Jmdjd3 promoted NLRP3 inflammasome regulation and exacerbated DSS-induced colitis in mice [78]. Genetic studies have also found that single nucleotide polymorphisms (SNP) of the *NLRP3* gene were highly associated with an increased susceptibility to CD [79]. These evidences strongly suggest that overactivation of the NLRP3 inflammasome leads to the overexpression of pro-inflammatory cytokines that promote inflammation and the development of IBD. 

In addition, a non-cytotoxic, acrylate-based NLRP3 inhibitor (INF39) was successfully developed that attenuated colitis through irreversible inhibition of the NLRP3 ATPase activity [80]. A follow-up study found that this direct NLRP3 inhibition by INF39 was more effective than caspase-1 or IL-1β suppression in ameliorating the effects of colitis [81]. This suggests that there is therapeutic potential for direct NLRP3 inhibition in the treatment of bowel inflammation [82].

### 2.6. The NLRP3 Inflammasome and CRC

Due to the effects of chronic inflammation that may be involved with tumorigenesis and the influence of the NLRP3 inflammasome on triggering and promoting inflammation; NLRP3 has been linked to many human malignancies, though its exact mechanisms remain unclear [83]. While NLRP3 is necessary for anticancer adaptive immune responses, its activation has also been related to several types of cancer due to the release of IL-1β and IL-18; including CRC, CAC, fibrosarcoma, transplantable tumour, lung cancer, thymoma, gastric cancer, hepatocellular carcinoma, breast tumours, head and neck cancers, prostate cancer, cervical cancer and central nervous system tumours [83,84]. 

A crucial study found that NLRP3 was overexpressed in CRC-positive tissue; adding that this high expression of NLRP3 was correlated with shorter lifespans and a poorer prognosis [85]. This notion was supported by a genetic study that showed that genetic alterations in the NF-κB axis influence prognosis of CRC patients [86]. Another study determined that NLRP3 expression was a prerequisite for epithelial-mesenchymal transition in colorectal cancer cells [87]; suggesting the role of NLRP3 in promoting cell migration and proliferation during CRC. 

## 3. Biological Features of the Aryl Hydrocarbon Receptor 

The aryl hydrocarbon receptor (AhR) is a member of the basic helix–loop–helix (bHLH) family of transcription factors. It is composed of three domains, each with its own functions. The N-terminal bHLH domain facilitates binding of AhR to the consensus regulatory sequences (5′-T/GCGTG-3′) of DNA [14,88]. The C-terminal variable domain promotes binding of AhR with its partner protein ARNT to form a heterodimeric complex [14,88]. The DNA-binding PER-ARNT-SIM (PAS) domain consists of PAS-A and PAS-B that is involved in secondary interactions with ARNT to ensure the formation of the heterozygous complex [14,88]. While it was discovered to have an integral role in the alleviation of toxic effects from environmental pollutants [88], recent studies have discovered its importance in the context of immunity and many cellular pathways. 

### 3.1. AhR Exogenous Ligands 

Most of the classical, high-affinity AhR ligands are environmental contaminants which consist of halogenated aromatic hydrocarbon (HAH), polycyclic aromatic hydrocarbons (PAH) and polychlorinated biphenyls (PCB); with 2,3,7,8-tetrachlorodibenzo-p-dioxin (TCDD) being one of the most potent and well-studied HAH ligands that can activate AhR receptors at picomolar concentrations [89]. HAHs are metabolically more stable [90] and are generally environmental waste resulting from industrial accidents or waste incineration products; which remain stable when extensively halogenated, but become potent AhR agonists when halogenated at lateral positions of the coplanar rings [91]. Chlorinated members of HAH agonists are known to elicit toxic responses that include epithelial hyperplasia, tumour promotion, teratogenesis, thymic involution and death [91]. PCBs are used in a wide variety of commercial products; including insulators, flame retardants and adhesives, due to their chemical stability and their potency are dependent on the halogens present on their benzene rings and the degree of substitution at their biphenyl bridge [91]. 3,3′,4,4′,5′-PentaCB is the most potent of the PCBs, but are still 100 times less potent than TCDD [91]. PAHs are metabolically more labile [90] and are commonly used in combustion processes; including chimney soot, charbroiled foods and smoke exhaust, which usually contain four or more conjugated benzene rings with a potency three to four-fold less than that of TCDD [91]. PAHs induce their own metabolisms due to their role as a substrate for the phase I and II xenobiotic metabolising enzymes [91].

### 3.2. AhR Endogenous Ligands 

#### 3.2.1. Tryptophan Metabolites

Tryptophan is one of the nine essential amino acids which acts as a precursor for many physiological functions in the body. Due to its aromaticity [91] and metabolism by spontaneous and enzyme-catalysed conversions and associated microbiota being a physiological source of numerous AhR agonists, it has been described to play a central role in many endogenous AhR ligands [92,93]. The main metabolic pathway (>90%) [92] of tryptophan in the body is the kynurenine (KYN) pathway via the enzyme isoforms indoleamine 2,3-dioxygenase (IDO1/2) and tryptophan 2,3-dioxygenase (TDO1/2) [92,94] driving in an inflammatory and carcinogenic pathway [93]. KYN is an intermediate-affinity AhR ligand with functions in Treg maturation and suppression of DCs [88]. This pathway leads to a positive feedback loop due to IDO being induced by AhR [95] and KYN metabolism can also lead to the production of kynurenic acid, xanthirenic acid and cinnabarinic acid, which are also potent AhR ligands [93].

In addition to KYN, the irradiation of L-tryptophan by visible light [94] or UV light will generate 6-formylindolo[3,2-*b*]carbazole (FICZ) through photolysis [93]. However, studies have found that FICZ can be generated via light-independent routes; such as through ROS via indole-3-acetalaldehyde [96]. FICZ is a high-affinity AhR agonist; similar potency to that of TCDD but it can be efficiently metabolised by the CYP1A1 enzyme [97], and can activate AhR at picomolar concentrations [94]. This contrast to TCDD allows FICZ to transiently regulate AhR activation and proposes a negative feedback loop for this pathway [98]. 

In addition, most indole derivatives are AhR agonists and are derived from the metabolism of tryptophan, which is an indole itself, by various bacteria from dietary sources [89]. The digestion of glucobrassicin from cruciferous vegetables; such as broccoli and Brussels sprouts, by stomach acid leads to the generation of indole-3-carbinol (I3C) and further degradation of this compound leads to the generation of indolo-[3,2-*b*]-carbazole (ICZ) and 3,3-diindolylmethane (DIM); both high-affinity AhR ligands with DIM being less potent than ICZ [91,94]. Lactobacilli have been found to produce indole-3-aldehyde (IAld) when under carbohydrate-starved conditions through catabolism of tryptophan via the ‘indole’ pathway [99]. Moreover, indole produced from *E.coli* metabolism via tryptophanase is broken down into indoxyl-3-sulfate (I3S) in the liver by the human sulfotransferase enzyme, SULT1A1 [88,100]. Furthermore, another study found that the intestinal microbiome was able to generate indole, tryptamine and indole-3-acetate from tryptophan, which all have AhR-mediated responses [101].

#### 3.2.2. Heme Metabolites 

Heme metabolites such as bilirubin and biliverdin have been found to have low-affinity agonistic AhR properties [97,98]. An early study by Sinal and Bend demonstrated the ability of bilirubin to induce Cyp1a1 gene expression in mouse hepatoma hepa 1c1c7 cells through an AhR-dependent pathway [102]. This was further supported in another study that suggested the potential for bilirubin as an AhR ligand due to the correlation between congenital jaundice Gunn rat and persistent CYP1A1 gene expression in these rats [103]. Homeostatic control of bilirubin serum concentration is important as this anti-oxidative compound has been implicated as a neurotoxin in newborns and Gilbert’s syndrome and Crigler–Najjar syndrome. These disorders arise due to a reduced or absent expression of the UGT1A1 enzyme, which is the major enzyme that detoxifies bilirubin [97]. The participation of CYP1A1, CYP1A2 and UGT1A in heme catabolism suggests a negative feedback loop on AhR activation due to the removal of physiologically relevant agonists [93]. Biliverdin and heme, being a precursor of bilirubin formation, was also shown to indirectly induce CYP1A1 gene expression through the heme metabolism pathway; while also inducing luciferase activity through AhR transformation and XRE binding in human and some rodent species, as well as competitively inhibit TCDD binding to AhR [102,103].

#### 3.2.3. Arachidonic Acid Derivatives

Arachidonic acid (AA) derivatives have been implicated to be endogenous ligands of AhR due to their metabolism by cytochrome P450 enzymes [104,105], which leads to the induction of CYP1A1 [106]. Cyclooxygenase-2 (COX-2) has been found to be induced following the TCDD-activation of AhR, which leads to the release of prostaglandins [107]; another potential ligand of AhR. Several prostaglandins were found to be weak agonists of AhR, with prostaglandin G2 being the most potent and capable of competitively displacing [3H]TCDD bound to AhR [108]. Lipoxin A4 was also found to be another AhR ligand, activating AhR in Hepa-1 cells, with lipoxin A4 competitively inhibiting the CYP1A1 enzyme [109]. However, due to their weak agonistic nature and difference in structure from other AhR ligands, AA may not be biologically relevant [91,93]. 

### 3.3. AhR Canonical Pathway

**Figure 3:**
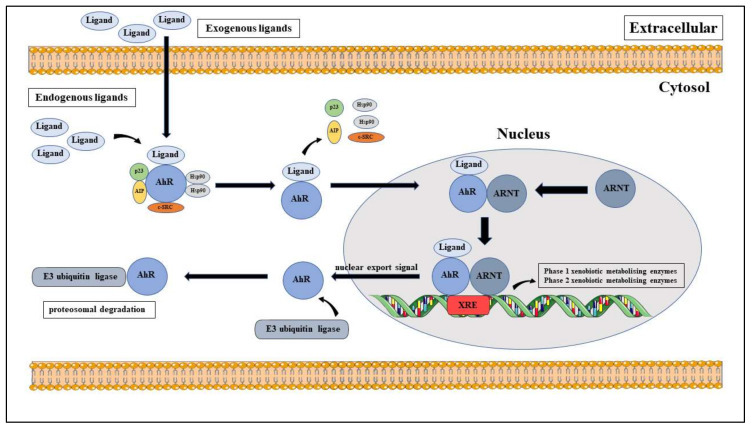
Canonical aryl hydrocarbon receptor (AhR) activation pathway.

The NLRP3 inflammasome is activated through multiple pathways; similarly, the activation of AhR can be categorised into the canonical and non-canonical activation pathways. The canonical pathway of AhR activation (Figure 3) begins with AhR forming an inactive, multi-protein complex in the cytoplasm with its 90 kDa chaperone protein, heat shock protein 90 (HSP90) and several scaffold protein; the co-chaperone, p23, the AhR interacting protein (AIP, also known as XAP2 or Ara9) [89,93,110], the kinome chaperone, Cdc37 and c-SRC protein kinase [111,112,113,114]. In this stable state, Hsp90 maintains the high-affinity ligand binding conformation and functional properties of AhR activation [93,115] whereas AIP prevents the degradation and ubiquitination of AhR [93,116]. Upon ligand binding, AhR undergoes a conformational change which exposes its nuclear translocation signal and releases it from its chaperone protein. However, Tsuji et al. contradicted this when they demonstrated that Hsp90 remains bound to this complex [117], to allow its translocation to the nucleus and heterodimerises with its nuclear partner, AhR nuclear translocator (ARNT), to form a ligand-AhR-ARNT complex [89,93,110]. Once in the nucleus, the ligand-AhR-ARNT complex binds to its cognate DNA consensus sequence (5′-TNGCGTG-3′); which is also known as the xenobiotic response element (XRE), located upstream of the Cytochrome P450 (CYP)1A1 and AhR repressor (AhRR) genes and lead to gene transcription of several metabolising enzymes. These include phase 1 xenobiotic-metabolising enzymes; such as *Cyp1a2* and *Cyp1b1*, and phase 2 xenobiotic-metabolising enzymes; such as glutathione-S-transferase A, NAD(P)H: quinone oxidoreductase 1, uridine 5′–diphosphate-glucuronosyltransferase 1A (UGT1A) and aldehyde dehydrogenase 3 [89,93,110]. Following its export from the nucleus, AhR undergoes proteasomal degradation through covalent binding with E3 ubiquitin ligase [88,118]. 

### 3.4. AhR Non-Canonical Pathway

The non-canonical AhR activation pathway is categorised by AhR-mediated gene transcription that does not contain XRE elements; through ligand-independent mechanisms, direct interactions with the protein and regulating phosphorylation events in the cytoplasm and nucleus [119]. There is evidence that shows cross-talk between AhR and the NF-κB subunit, RelB through the binding with the XRE-like sequence, CC-chemokine ligand 1 (CCL1), located at the IL-8 gene regulatory region [120]. AhR has also been shown to directly interact with the oestrogen receptor by acting as an E3 ubiquitin ligase and facilitating its degradation [121,122,123]. Several studies have also shown that AhR can directly bind with the retinoblastoma receptor and the E2F promoter to form a complex that leads suppression of S phase-specific genes and the arrest of the cell cycle at the G1 phase [124,125,126]. In addition, Kruppel-like Factor 6 (KLF6); a tumour suppressor through regulation of the p21^Cip1^ cyclin-dependent kinase inhibitor [127,128], has been identified as a novel DNA-binding partner of AhR to express inducible-TCDD when bound at the non-consensus XRE [129,130]. 

### 3.5. Regulation of AhR Activation 

In order to maintain a healthy cell physiology, the activation of AhR has to be regulated. There are various ways that maintain this homeostasis. Firstly, following its export out of the nucleus by a nuclear export signal (NES), the ligand-activated AhR is broken down by the 26S ubiquitin-proteosome system [119,131]. Secondly, the phase I and II xenobiotic metabolising enzymes are responsible for an auto-regulatory feedback loop with AhR activation [94]. The upstream activation signal of these enzymes by AhR leads to the degradation of AhR ligands and prevent prolonged AhR activation [94,110]. Thirdly, the activation of AhR also leads to the transcription of the AhRR, which is an AhR inhibitor. AhRR inhibits AhR activity by direct competition with AhR for binding with ARNT; in which AhRR has a higher affinity for ARNT than AhR, thus interfering with the AhR-ARNT complex to prevent its binding with DNA and leads to AhR proteosomal degradation [94,110,119]. It should be noted that AhRR expression does not always correspond with CYP1A1 induction; even though AhR-mediated transcription leads to the expression of both AhRR and CYP1A1 [132]. This indicates AhRR may also be involved with other signalling pathways and have other cellular functions, since the *Ahrr* genes contain binding sites that are recognised by the NF-κB and zinc-finger transcription factors of the Sp1 family [132]. Lastly, the mono-ADP-ribosyltransferase, TCDD-inducible Poly ADP-Ribose Polymerase (TiPARP), has been shown in several studies to negatively regulate AhR transactivation; comparably with AhRR but utilising different mechanisms, through a negative feedback loop in the AhR signalling pathway and modulating its expression [133,134,135,136].

### 3.6. AhR in Inflammation-Related Diseases

A prominent case of AhR influencing immunity is the failed assassination attempt by TCDD poisoning of the 2004 Ukraine president candidate, Victor Yushchenko [137]. TCDD diffuses freely across the plasma membrane and exerts its toxic properties when bound to AhR; which lead to Viktor developing symptoms of acute pancreatitis and chloracne at a concentration of 50,000-fold to the normal TCDD concentration of the general population [137,138]. This incident has led to extensive research on AhR to elucidate its roles in immune system disorders; such as in microbial infections, major depressive disorder, multiple sclerosis, congenital nystagmus, inflammatory diseases, autoimmune diseases and a wide range of cancers [139,140]. The link between AhR and the gut is extensively studied due to AhR being highly expressed in the gut [140]. There is increasing evidence of AhR dampening the immune response in inflammatory diseases that include types of IBD; such as CD and UC [89] through its involvement with NLRP3. A recent study had determined a link between NLRP3 and AhR in preschool children. This study found that PAH exposure, an environmental ligand of AhR, lead to an increased expression of several pro-inflammatory cytokines regulated by the NLRP3 inflammasome, which lead to cytokine storms developing in these children [140]. Another study by Postal et al. found a role for AhR in maintaining intestinal barrier integrity; concluding that AhR elicited a protective effect by cross-talking with several signalling pathways that promoted the maintenance of tight junctions [141].

## 4. Therapeutic Application of AhR in IBD and CAC

### 4.1. AhR Acts as a Natural NLRP3 Inhibitor

While PTMs play an important role in the regulation of the NLRP3 inflammasome activation, it can also be regulated through modifications at the transcription level with NF-κB. As previously mentioned, NF-κB plays a central role in the priming step and leads to the formation of pro-IL-1β, pro-IL-18 and the NLRP3 protein; which is needed for the activation of the NLRP3 inflammasome, following stimulation of the TLRs by Signal 1 [141,142]. Afonina et al. have identified several negative regulators of the NF-κB pathway that inhibit NLRP3 inflammasome activation, which may be the potential therapeutic target for inflammatory and autoimmune diseases [143]. 

Besides NF-κB, AhR has been shown to play a part in the innate immune response; such as in *Listeria monocytogenes* (LM) infections, where Kimura et al. revealed that AhR knockout macrophages had increased expression of the pro-inflammatory cytokines, IL-6 and TNF-α, and promoted caspase-3 activation; which lead to decreased macrophage survival and increased susceptibility to listeriosis [144]. The study further described that AhR also induces the expression of p40^phox^ and the production of ROS, which further enhanced bacterial clearance during LM infection [144]. AhR and the NLRP3 inflammasome has been implicated in a study by Jia et al., which determined that their link was associated with the pathogenesis of acute myeloid leukaemia and an imbalance in the T_H_ population; through measuring AhR and NLRP3 molecules in the bone marrow and peripheral mononuclear cells of newly diagnosed and remission patients [145]. Importantly, the myeloid-specific microRNA, miR-223 was identified as a negative regulator of the NLRP3 inflammasome through transcriptional control at *NLRP3* 3′-UTR [146,147]. Coincidentally, Ogando et al. determined that miR-223 acts as a regulator of the AhR/ARNT pathway and its expression leads to the down-regulation of the AhR/ARNT and increased pro-inflammatory cytokine production in rheumatoid arthritis patients [148]. Interestingly, miR-223 is a common regulator of both the AhR/ARNT and NLRP3 activation pathways.

The link between AhR and NF-κB was identified by Tian et al. who described that AhR and NF-κB RelA directly interact with each other by physical association and functional modulation following dissociation from their respective regulatory mechanisms after stimulation by their respective activation signals [149]. The study also proposes two mechanism models that may explain their mutual repression following their interaction; which are, the formation of an inactive complex or regulation by the transcription mediator p300/CREB-binding protein (CBP) that leads to competitive binding between ligand-AhR/ARNT complexes and RelA for p300/CBP [149]. This notion is supported by another study that determined NF-κB and the glucocorticoid receptor mutually repressed each other through the co-activators, CBP and steroid receptor coactivator-1 (SRC-1) [150]. In addition to NF-κB RelA, NF-κB RelB was also found to crosstalk with AhR [151,152]. Another link between AhR and the NF-κB pathway was demonstrated by Wang et al., in which they exemplified that quaking plays a role in inhibiting innate immune responses via regulation of the AhR/STAT1–NF-κB pathway [153].

**Figure 4:**
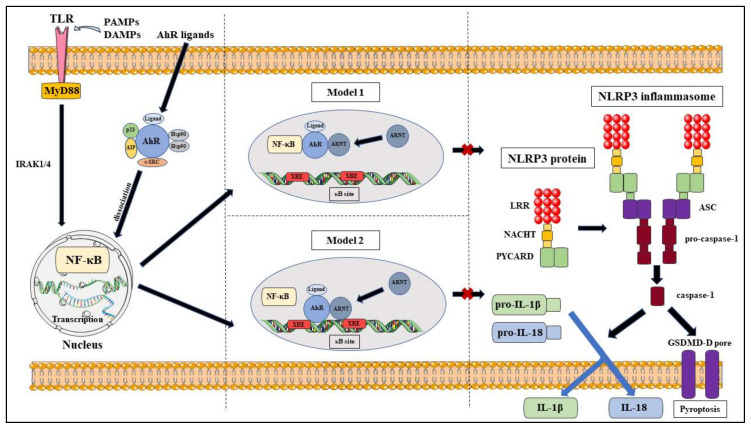
Proposed models of NLRP3 inflammasome inhibition via AhR pathway.

The interactions between AhR and NF-κB lead to the hypothesis that AhR can also regulate NLRP3 inflammasome activity via the NF-κB pathway (Figure 4). A critical study by Huai et al. determined that AhR negatively regulates NLRP3 inflammasome activation at the transcriptional level via the NF-κB pathway by using TCDD-treated, LPS-induced mouse peritoneal macrophages to show reduced Nlrp3 expression at the protein and mRNA level [154]. They also identified that the inhibitory action was due to AhR binding to XRE sequences at the two NF-κB binding sites located in the NLRP3 promoter region [154]. Their proposed mechanism models were that NLRP3 acted as the rate-limiting element in its activation and that either AhR competed with NF-κB for binding sites or that AhR ‘tethered’ NF-κB to prevent the transcriptional process [154]. AhR has also been shown to negatively regulate LPS-induced inflammatory responses through its interactions with the NF-κB pathway via Stat1 to inhibit IL-6 activities [155] and via plasminogen activator inhibitor-2 (Pai-2) to inhibit IL-1β expression [156]. In addition to that, Zhu et al. also illustrated that the NF-κB pathway mediated LPS-induced AhR expression in macrophages [157]. However, it should be noted that a controversial study found that indoxyl sulfate-induced the expression of macrophage IL-1β via AhR-NF-κB/MAPK cascade, while bypassing NLRP3 activation [158]. This result may be specific to sustained chronic inflammation in the kidneys and may utilise different mechanisms than that of chronic colitis. 

### 4.2. Role of Mouse Models in the Investigation of AhR in Maintaining Intestinal Homeostasis

AhR and its effects on the NLRP3 inflammasome and colitis have been predominantly studied using wild-type (AhR^+/+^), AhR knockout (AhR^−/−^) and heterozygous (AhR^−/+^) mouse models that have been chemically induced with intestinal inflammation; by using 2,4,6-trinitrobenzene sulphonic acid (TNBS) or dextran sulphate sodium (DSS), and CAC; by using azoxymethane (AOM or AOM/DSS) [70]. Several studies have determined that AhR exhibits protective effects during intestinal inflammation; with TCDD-treated and FICZ-treated mice ameliorating colitis severity, which were determined from corrected weight loss, reduced colitis symptoms and recover periods [159,160,161,162,163]. These reversed colitis effects were due to a change in their cytokine environment, with AhR-activated mice having a reduction in the expression of pro-inflammatory mediators; such as IL-6, IL-12, IL-17, IFN-c, MCP-1, exotaxin-1 and TNF-α, but an increase in IL-22 and TGF-β [159,162,163]. The results from Monteleone et al. supports this notion with FICZ-treated TNBS-induced mice showing reversed colitis symptoms while also showing that colitis symptoms were enhanced when the mice were treated with AhR antagonists [164]. They further determined a link between FICZ and IL-22; with FICZ increasing the expression of IL-22, and anti-IL-22 leading to a reduction in the anti-inflammatory effects of FICZ [164]. Takamura et al. proposed that the inhibition of colitis by AhR may be due to the production of PGE2; and that the inhibition of PGE2 reduced the inhibitory effects of AhR on colitis [165]. Furumatsu et al. found that AhR knockout mice displayed more severe DSS-induced colitis when compared to the C57BL/6J wild-type mice [166]. In accordance to that, Arsenescu et al. proclaimed that wild-type mice developed severe colitis and AhR knockout mice died early in inflammation; but heterozygous mice exhibited little change in intestinal histology and had good clinical outcomes [167]. These heterozygous mice had reduced expression of TNF-α as compared to the other two models [167]. The disparity of results seen in these mouse models suggests more investigations to be done in order to achieve a greater insight into the mechanisms involved in the protective roles of AhR in maintaining intestinal homeostasis.

### 4.3. Therapeutic Potential of AhR as A Natural NLRP3 Inflammasome Inhibitor 

The understanding of the signalling pathways of AhR and the NLRP3 inflammasome and how they intertwine with each other is important in assessing the therapeutic potential of AhR in the treatment of IBD and CAC (Table 1). The abundance of natural AhR ligands found in the environment provides a positive outlook for future therapeutic agents that utilise AhR as an immunomodulator through its inhibitory effects on NLRP3 inflammasome activation. We have identified some NLRP3 inhibitors that utilise the AhR pathway have been described in an experimental context and have been demonstrated to be effective.

#### 4.3.1. Therapeutic Potential of AhR as A Natural NLRP3 Inflammasome Inhibitor in Bowel Inflammation

Norisoboldine (NOR) is the primary isoquinoline alkaloid constituent of Radix Linderae; the dry root of *Lindera aggregata* (Sims) Kosterm (*L. strychnifolia* Vill), which is commonly used in Chinese medicine in treating a variety of ailments, which include constipation, polyuria, dyspepsia, acute and chronic colitis, chest and abdominal pain and rheumatism palsy [168,169]. NOR has been shown to be a natural AhR ligand by Qi et al. through its up-regulation of CYP1A1 expression, promotion of AhR nuclear translocation after dissociation of the AhR/Hsp90 complex and transcriptional activity of AhR/ARNT via DNA binding to the XRE region in THP-1 cells [169]. Studies also reveal that NOR promotes T_reg_ cell differentiation from naïve T-cells in-vitro and increased its immunosuppressive effects on T_eff_ cells through inducing apoptotic events and inhibiting T_h_1 and T_h_17 differentiation [170,171]. In the context of IBD, NOR has also been demonstrated to reduce NLRP3, ASC and caspase-1 expression in TNBS-induced mice, which could imply that NOR has inhibitory effects on NLRP3 inflammasome activation through an AhR pathway [169]. The study further found a link between NOR and Nrf2, a nuclear transcriptional factor of Cap’n’ Collar family that can inhibit NLRP3 priming [172], as the mechanism for NOR-induced inhibition of NLRP3 inflammasome activation; where NOR up-regulated the expression of Nrf2 but down-regulated the production of ROS signals in THP-1 cells [169]. Concurrently, Qi et al. also demonstrated that NOR was able to produce anti-UC effects through the suppression of glycolysis and promotion of T_reg_ cell differentiation in hypoxic microenvironments via regulation of the NAD^+^/SIRT1/SUV39H1/H3K9me3 signalling pathway [170].

Cardamonin (20,40-dihydroxy-60-methoxychalcone) is a chalcone that is the main flavonoid isolated from the dry, mature seeds of the medicinal herb, *Alpinia katsumadai* Hayata [173,174,175]. It has been used for thousands of years as a traditional Chinese medicine due to its anti-inflammatory and anti-tumour properties [173,174,175]. The anti-inflammatory activity of cardamonin was studied by Wang et al., in which they demonstrated that cardamonin inhibited NLRP3 inflammasome expression and activation in mice colon with TNBS- and DSS-induced colitis and LPS- and ATP-stimulated macrophages [175]. They further displayed that AhR and Nrf2 were also influenced by cardamonin as evidenced by the up-regulation of CYP1A1 mRNA and protein levels, and the up-regulation of Nrf2 protein levels in THP-1 cells, respectively [175]. The AhR/Nrf2 signals produced by cardamonin would then activate NQO1, which has inhibitory effects in NLRP3 inflammasome priming, which lead to a reduction in pro-inflammatory cytokines and produce anti-colitis effects [172,175]. Further studies by Wang et al. revealed that cardamonin acts a broad-spectrum and specific inhibitor of the NLRP3 inflammasome through their mouse models which show that cardamonin increased the survival rate of mice with LPS-induced septic shock and down-regulated expression of TNF-α and IL-1β [174]. 

#### 4.3.2. Therapeutic Potential of AhR as a Natural NLRP3 Inflammasome Inhibitor in CAC

An early study discovered the link between AhR and colon neoplasia when they characterised the association of AhR with the human colon adenocarcinoma cell line LS180 [176]. This link was also observed in a study that utilised mouse models with sporadic colon cancer that were fed a Western-style diet; in which oxidative stress response and immune dysregulation via Nrf2 and AhR pathways were induced [177]. Another study using AOM/DSS-induced mouse models found that the absence of AhR increased their susceptibility in colorectal tumorigenesis. The same study also found that the AhR ligand, I3C protected against the development of colorectal tumours and is AhR-dependent [178]. The association between the NLRP3 inflammasome and AhR in CAC was shown in a study by Ikuta et al., in which cecal tumours developed in AhR-null mice in an ASC-dependent manner. This same study also determined that AhR plays dual tumour suppressor roles; as a regulator of intestine anti-inflammation and β-catenin degradation [179]. Cardamonin was also shown to have protective effects against tumorigenesis via AhR. The anti-tumour activity of cardamonin was explored by Wang et al., which indicated that cardamonin exerts anti-gastric cancer properties by down-regulating signal transducer and activator of transcription 3 (STAT3) via the LncRNA-PVT1-STAT3 signalling pathway in human gastric cells, AGS [173]. 

While NOR and cardamonin have been described to naturally inhibit NLRP3 activation and reducing the inflammatory effects via AhR, more studies should be conducted to expand the variety of these natural inhibitors. 

## 5. Conclusions

It is clear that there is substantial evidence that supports the role that AhR plays as a natural inhibitor of the NLRP3 inflammasome at the transcriptional level. AhR acts as the primary link between nature and the immune system due to the abundance of its ligands found in the environment and our daily consumptions. Indeed, its link to the NLRP3 inflammasome also validates the role AhR can play in the treatment of colitis in IBD and CAC. The exact mechanisms behind the crosstalk of these signalling pathways are under vigorous investigation, however, the current models proposed for this mechanism are still matters of conjecture. Promisingly there are successful therapeutic agents determined to be AhR agonists that treat TNBS- and DSS-induced colitis via the NLRP3 inflammasome but they are still far from many. Thus, follow-up studies are essential to look further into deciphering the precise mechanisms of NLRP3 inflammasome inhibition by AhR. This will open a pathway for the use of AhR-dependent therapeutic agents; as an alternative treatment method of IBD and CAC, that may prove to be more potent and possibly a safer option as compared to current treatment methods. 

## Figures and Tables

**Table 1:** Roles of the NLRP3 inflammasome and AhR in inflammation and cancer.

Pathways	Mechanism	Ref.
AhR/STAT1–NF-κB axis	Quaking inhibits NF-κB transcriptional activity and prevents NLRP3 inflammation via AhR-dependent manner	[153]
NAD^+^/SIRT1/SUV39H1/H3K9me3 axis	Ameliorates colitis through promotion of T_reg_ differentiation	[170]
LncRNA-PVT1-STAT3 axis	Inhibits STAT3 via LncRNA-PVT1 from promoting signals for cancer inflammation	[173]
AhR/Nrf2/NQO1 axis	AhR up-regulates Nrf2 and NQO1 levels in the colon and prevents NLRP3 inflammasome activation	[175]
Fibronectin/integrin β1/FAK axis	Promotes cancer metastasis through ANRT down-regulation	[180]

## Abbreviations

AA	Arachidonic acid
AhR	Aryl hydrocarbon receptor
AhRR	Aryl hydrocarbon receptor repressor
ARNT	Aryl hydrocarbon receptor nuclear translocator
bHLH	Basic helix–loop–helix
CAC	Colitis-associated colorectal cancer
CD	Crohn’s disease
CRC	Colorectal cancer
CYP	Cytochrome P450
DAMP	Danger-associated molecular pattern
DSS	Dextran sulfate sodium
FICZ	6-formylindolo(3,2b)carbazole
GAPDH	Glyceraldehyde 3-phosphate dehydrogenase
GSDMD-D	Gasdermin D
HAH	Halogenated aromatic hydrocarbon
HSP90	Heat shock protein 90
IBD	Inflammatory bowel disease
KYN	Kynurenine
LPS	Lipopolysaccharide
LRR	Leucine-rich repeat
MAVS	Mitochondrial antiviral-signalling protein
MyD88	Myeloid differentiation primary response 88
NEK7	NIMA-related kinase 7
NF-κB	Nuclear factor kappa-light-chain-enhancer of activated B cells
NLR	Nod-like receptor
NLRP3	Nod-like receptor family pyrin domain containing 3
NOR	Norisoboldine
PAH	Polycyclic aromatic hydrocarbons
PAMP	Pathogen-associated molecular pattern
PRR	Pathogen recognition receptor
PTM	Post-translational modification
TCDD	2,3,7,8-Tetrachlorodibenzo-p-dioxin
TLR	Toll-like receptor
TNBS	2,4,6-trinitrobenzene sulfonic acid
UC	Ulcerative colitis
XRE	Xenobiotic responsive element

## References

[1] Palazon-Riquelme P., Lopez-Castejon G. (2018). The inflammasomes, immune guardians at defence barriers. Immunol..

[2] Pulusu S.S.R., Lawrance I. (2017). Dysplasia and colorectal cancer surveillance in inflammatory bowel disease. Expert Rev. Gastroenterol. Hepatol..

[3] Lutgens M.W.M.D., Vleggaar F.P., Van Oijen M.G., Van Der Heijden G.J.M.G., Siersema P.D., Oldenburg B. (2013). Declining Risk of Colorectal Cancer in Inflammatory Bowel Disease. Inflamm. Bowel Dis..

[4] Burisch J., Munkholm P. (2015). The epidemiology of inflammatory bowel disease. Scand. J. Gastroenterol..

[5] Ou B., Zhao J., Guan S., Lu A. (2015). Survival of Colorectal Cancer in Patients With or Without Inflammatory Bowel Disease: A Meta-Analysis. Dig. Dis. Sci..

[6] Dulai P.S., Sandborn W.J., Gupta S. (2016). Colorectal Cancer and Dysplasia in Inflammatory Bowel Disease: A Review of Disease Epidemiology, Pathophysiology, and Management. Cancer Prev. Res..

[7] Ananda S.S., Kosmider S., Tran B., Field K., Jones I., Skinner I., Guerrieri M., Chapman M., Gibbs P. (2015). The rapidly escalating cost of treating colorectal cancer in Australia. Asia-Pacific, J. Clin. Oncol..

[8] Lew J.-B., John D.J.B.S., Xu X.-M.E., Greuter M.J., Caruana M., Cenin D.R., He E., Saville M., Grogan P., Coupé V.M.H. (2017). Long-term evaluation of benefits, harms, and cost-effectiveness of the National Bowel Cancer Screening Program in Australia: A modelling study. Lancet Public Heal..

[9] Man S.M., Kanneganti T.-D. (2015). Regulation of inflammasome activation. Immunol. Rev..

[10] Martinon F., Burns K., Tschopp J. (2002). The inflammasome: A molecular platform triggering activation of inflammatory caspases and processing of proIL-beta. Mol Cell..

[11] He Y., Hara H., Nunez G. (2016). Mechanism and Regulation of NLRP3 Inflammasome Activation. Trends Biochem. Sci..

[12] Perera A.P., Sajnani K., Dickinson J., Eri R., Körner H. (2018). NLRP3 inflammasome in colitis and colitis-associated colorectal cancer. Mamm. Genome.

[13] Guo H., Callaway J.B., Ting J.P.-Y. (2015). Inflammasomes: Mechanism of action, role in disease, and therapeutics. Nat. Med..

[14] Feng S., Cao Z., Wang X. (2013). Role of aryl hydrocarbon receptor in cancer. Biochim. et Biophys. Acta (BBA) - Bioenerg..

[15] Jo E.-K., Kim J.K., Shin N.-M., Sasakawa C. (2015). Molecular mechanisms regulating NLRP3 inflammasome activation. Cell. Mol. Immunol..

[16] Yang Y., Wang H., Kouadir M., Song H., Shi F. (2019). Recent advances in the mechanisms of NLRP3 inflammasome activation and its inhibitors. Cell Death Dis..

[17] Bauernfeind F.G., Horvath G.L., Stutz A., Alnemri E.S., Macdonald K., Speert D., Fernandes-Alnemri T., Wu J., Monks B.G., Fitzgerald K.A. (2009). Cutting edge: NF-kappaB activating pattern recognition and cytokine receptors license NLRP3 inflammasome activation by regulating NLRP3 expression. J. Immunol..

[18] Groslambert M., Py B. (2018). Spotlight on the NLRP3 inflammasome pathway. J. Inflamm. Res..

[19] Tschopp J., Schroder K. (2010). NLRP3 inflammasome activation: The convergence of multiple signalling pathways on ROS production?. Nat. Rev. Immunol..

[20] Muñoz-Planillo R., Kuffa P., Martínez-Colón G., Smith B.L., Rajendiran T.M., Nunez G. (2013). K⁺ efflux is the common trigger of NLRP3 inflammasome activation by bacterial toxins and particulate matter. Immun..

[21] Gong T., Yang Y., Jin T., Jiang W., Zhou R. (2018). Orchestration of NLRP3 Inflammasome Activation by Ion Fluxes. Trends Immunol..

[22] Tang T., Lang X., Xu C.-F., Wang X., Gong T., Yang Y., Cui J., Bai L., Wang J., Jiang W. (2017). CLICs-dependent chloride efflux is an essential and proximal upstream event for NLRP3 inflammasome activation. Nat. Commun..

[23] Daniels M., Rivers-Auty J., Schilling T., Spencer N.G., Watremez W., Fasolino V., Booth S.J., White C., Baldwin A.G., Freeman S. (2016). Fenamate NSAIDs inhibit the NLRP3 inflammasome and protect against Alzheimer’s disease in rodent models. Nat. Commun..

[24] Tait S.W., Green D.R. (2012). Mitochondria and cell signalling. J. Cell Sci..

[25] Zhou R., Yazdi A.S., Menu P., Tschopp J. (2010). A role for mitochondria in NLRP3 inflammasome activation. Nature.

[26] Wen H., Gris D., Lei Y.L., Jha S., Zhang L., Huang M.T.-H., Brickey W.J., Ting J.P.-Y. (2011). Fatty acid-induced NLRP3-ASC inflammasome activation interferes with insulin signaling. Nat. Immunol..

[27] Subramanian N., Natarajan K., Clatworthy M.R., Wang Z., Germain R.N. (2013). The adaptor MAVS promotes NLRP3 mitochondrial localization and inflammasome activation. Cell.

[28] Orlowski G.M., Colbert J.D., Sharma S., Bogyo M., Robertson S.A., Rock K.L. (2015). Multiple Cathepsins Promote Pro-IL-1β Synthesis and NLRP3-Mediated IL-1β Activation. J. Immunol..

[29] Duewell P., Kono H., Rayner K.J., Sirois C.M., Vladimer G., Bauernfeind F.G., Abela G.S., Franchi L., Núñez G., Schnurr M. (2010). NLRP3 inflammasomes are required for atherogenesis and activated by cholesterol crystals. Nat..

[30] Newman Z.L., Leppla S.H., Moayeri M. (2009). CA-074Me Protection against Anthrax Lethal Toxin. Infect. Immun..

[31] Gupta R., Ghosh S., Monks B., DeOliveira R.B., Tzeng T.-C., Kalantari P., Nandy A., Bhattacharjee B., Chan J., Ferreira F. (2014). RNA and β-Hemolysin of Group B Streptococcus Induce Interleukin-1β (IL-1β) by Activating NLRP3 Inflammasomes in Mouse Macrophages*. J. Boil. Chem..

[32] Barlan A.U., Griffin T., McGuire K.A., Wiethoff C.M. (2010). Adenovirus Membrane Penetration Activates the NLRP3 Inflammasome. J. Virol..

[33] Dostert C., Guarda G., Romero J.F., Menu P., Groß O., Tardivel A., Suvà M.-L., Stehle J.-C., Kopf M., Stamenkovic I. (2009). Malarial Hemozoin Is a Nalp3 Inflammasome Activating Danger Signal. PLoS ONE.

[34] Netea M.G., Nold-Petry C.A., Nold M.F., Joosten L.A.B., Opitz B., Van Der Meer J.H.M., Van De Veerdonk F.L., Ferwerda G., Heinhuis B., Devesa I. (2009). Differential requirement for the activation of the inflammasome for processing and release of IL-1β in monocytes and macrophages. Blood.

[35] Elliott E., Sutterwala F.S. (2016). Monocytes Take Their Own Path to IL-1β. Immun..

[36] Gaidt M.M., Hornung V. (2017). Alternative inflammasome activation enables IL-1β release from living cells. Curr. Opin. Immunol..

[37] Antonopoulos C., Russo H.M., El Sanadi C., Martin B.N., Li X., Kaiser W.J., Mocarski E.S., Dubyak G.R. (2015). Caspase-8 as an Effector and Regulator of NLRP3 Inflammasome Signaling. J. Boil. Chem..

[38] Chung H., Vilaysane A., Lau A., Stahl M., Morampudi V., Bondzi-Simpson A., Platnich J.A., Bracey N., French M.-C., Beck P.L. (2016). NLRP3 regulates a non-canonical platform for caspase-8 activation during epithelial cell apoptosis. Cell Death Differ..

[39] Gringhuis S.I., Kaptein T.M.A., Wevers B., Theelen B., Van Der Vlist M., Boekhout T., Geijtenbeek T.B.H. (2012). Dectin-1 is an extracellular pathogen sensor for the induction and processing of IL-1β via a noncanonical caspase-8 inflammasome. Nat. Immunol..

[40] Gaidt M.M., Ebert T.S., Chauhan D., Schmidt T., Schmid-Burgk J.L., Rapino F., Robertson A.A., Cooper M.A., Graf T., Hornung V. (2016). Human Monocytes Engage an Alternative Inflammasome Pathway. Immun..

[41] Song N., Li T. (2018). Regulation of NLRP3 Inflammasome by Phosphorylation. Front. Immunol..

[42] Juliana C., Fernandes-Alnemri T., Kang S., Farias A., Qin F., Alnemri E.S. (2012). Non-transcriptional Priming and Deubiquitination Regulate NLRP3 Inflammasome Activation. J. Boil. Chem..

[43] Gong Y.-N., Wang X., Wang J., Yang Z., Li S., Yang J., Liu L., Lei X., Shao F. (2010). Chemical probing reveals insights into the signaling mechanism of inflammasome activation. Cell Res..

[44] Palazon-Riquelme P., Worboys J.D., Green J., Valera A., Martín-Sánchez F., Pellegrini C., Brough D., Lopez-Castejon G. (2018). USP7 and USP47 deubiquitinases regulate NLRP3 inflammasome activation. EMBO Rep..

[45] Py B., Kim M.-S., Vakifahmetoglu-Norberg H., Yuan J. (2013). Deubiquitination of NLRP3 by BRCC3 Critically Regulates Inflammasome Activity. Mol. Cell.

[46] Sandall C.F., Macdonald J.A. (2019). Effects of phosphorylation on the NLRP3 inflammasome. Arch. Biochem. Biophys..

[47] Song N., Liu Z.-S., Xue W., Bai Z.-F., Wang Q.-Y., Dai J., Liu X., Huang Y.-J., Cai H., Zhan X.-Y. (2017). NLRP3 Phosphorylation Is an Essential Priming Event for Inflammasome Activation. Mol. Cell.

[48] Humphries F., Bergin R., Jackson R., Delagic N., Wang B., Yang S., Dubois A.V., Ingram R., Moynagh P. (2018). The E3 ubiquitin ligase Pellino2 mediates priming of the NLRP3 inflammasome. Nat. Commun..

[49] Martin B.N., Wang C., Willette-Brown J., Herjan T., Gulen M.F., Zhou H., Bulek K., Franchi L., Sato T., Alnemri E.S. (2014). IKKα negatively regulates ASC-dependent inflammasome activation. Nat. Commun..

[50] Lee S.H., Toth Z., Wong L.-Y., Brulois K., Nguyen J., Lee J.-Y., Zandi E., Jung J.U. (2012). Novel Phosphorylations of IKKγ/NEMO. mBio.

[51] Ghonime M.G., Shamaa O.R., Das S., Eldomany R.A., Fernandes-Alnemri T., Alnemri E.S., Gavrilin M.A., Wewers M.D. (2014). Inflammasome priming by lipopolysaccharide is dependent upon ERK signaling and proteasome function. J. Immunol..

[52] D’Espessailles A., Mora Y.A., Fuentes C., Cifuentes M. (2018). Calcium-sensing receptor activates the NLRP3 inflammasome in LS14 preadipocytes mediated by ERK1/2 signaling. J. Cell. Physiol..

[53] Li D., Ren W., Jiang Z., Zhu L. (2018). Regulation of the NLRP3 inflammasome and macrophage pyroptosis by the p38 MAPK signaling pathway in a mouse model of acute lung injury. Mol. Med. Rep..

[54] Guo H., Sun L., Ling S., Xu J.-W. (2018). Levistilide A Ameliorates NLRP3 Expression Involving the Syk-p38/JNK Pathway and Peripheral Obliterans in Rats. Mediat. Inflamm..

[55] Rajamäki K., Mäyränpää M.I., Risco A., Tuimala J., Nurmi K., Cuenda A., Eklund K.K., Öörni K., Kovanen P.T. (2016). p38δ MAPK. Arter. Thromb. Vasc. Boil..

[56] Haggadone M.D., Grailer J.J., Fattahi F., Zetoune F.S., Ward P.A. (2016). Bidirectional Crosstalk between C5a Receptors and the NLRP3 Inflammasome in Macrophages and Monocytes. Mediat. Inflamm..

[57] Yu S., Wang D., Huang L., Zhang Y., Luo R., Adah D., Tang Y., Zhao K., Lu B. (2019). The complement receptor C5aR2 promotes protein kinase R expression and contributes to NLRP3 inflammasome activation and HMGB1 release from macrophages. J. Boil. Chem..

[58] Lu B., Nakamura T., Inouye K., Li J., Tang Y., Lundbäck P., Valdés-Ferrer S., Olofsson P.S., Kalb T., Roth J. (2012). Novel role of PKR in inflammasome activation and HMGB1 release. Nat..

[59] Basak C., Pathak S., Bhattacharyya A., Mandal D., Pathak S., Kundu M. (2004). NF-κB- and C/EBPβ-driven Interleukin-1β Gene Expression and PAK1-mediated Caspase-1 Activation Play Essential Roles in Interleukin-1β Release fromHelicobacter pyloriLipopolysaccharide-stimulated Macrophages. J. Boil. Chem..

[60] Groß C.J., Groß O. (2016). PKA Has the Gall to Oppose NLRP3. Immun..

[61] Guo C., Xie S., Chi Z., Zhang J., Liu Y., Zhang L., Zheng M., Zhang X., Xia D., Ke Y. (2016). Bile Acids Control Inflammation and Metabolic Disorder through Inhibition of NLRP3 Inflammasome. Immun..

[62] Mortimer L., Moreau F., Macdonald J.A., Chadee K. (2016). NLRP3 inflammasome inhibition is disrupted in a group of auto-inflammatory disease CAPS mutations. Nat. Immunol..

[63] Zhang Z., Meszaros G., He W.-T., Xu Y., Magliarelli H.D.F., Mailly L., Mihlan M., Liu Y., Gámez M.P., Goginashvili A. (2017). Protein kinase D at the Golgi controls NLRP3 inflammasome activation. J. Exp. Med..

[64] Kawashima A., Karasawa T., Tago K., Kimura H., Kamata R., Usui-Kawanishi F., Watanabe S., Ohta S., Funakoshi-Tago M., Yanagisawa K. (2017). ARIH2 Ubiquitinates NLRP3 and Negatively Regulates NLRP3 Inflammasome Activation in Macrophages. J. Immunol..

[65] Song H., Liu B., Huai W., Yu Z., Wang W., Zhao J., Han L., Jiang G., Zhang L., Gao C. (2016). The E3 ubiquitin ligase TRIM31 attenuates NLRP3 inflammasome activation by promoting proteasomal degradation of NLRP3. Nat. Commun..

[66] Mao K., Chen S., Wang Y., Zeng Y., Ma Y., Hu Y., Zhang H., Sun S., Wu X., Meng G. (2015). β-arrestin1 Is Critical for the Full Activation of NLRP3 and NLRC4 Inflammasomes. J. Immunol..

[67] Yan Y., Jiang W., Liu L., Wang X., Ding C., Tian Z., Zhou R. (2015). Dopamine Controls Systemic Inflammation through Inhibition of NLRP3 Inflammasome. Cell.

[68] Stutz A., Kolbe C.-C., Stahl R., Horvath G.L., Franklin B.S., Van Ray O., Brinkschulte R., Geyer M., Meissner F., Latz E. (2017). NLRP3 inflammasome assembly is regulated by phosphorylation of the pyrin domain. J. Exp. Med..

[69] Spalinger M.R., Kasper S., Gottier C., Lang S., Atrott K., Vavricka S.R., Scharl S., Raselli T., Frey-Wagner I., Gutte P.M. (2016). NLRP3 tyrosine phosphorylation is controlled by protein tyrosine phosphatase PTPN22. J. Clin. Investig..

[70] Perera A.P., Fernando R., Shinde T., Gundamaraju R., Southam B., Sohal S.S., Robertson A.A.B., Schroder K., Kunde D., Eri R. (2018). MCC950, a specific small molecule inhibitor of NLRP3 inflammasome attenuates colonic inflammation in spontaneous colitis mice. Sci. Rep..

[71] Gong Z., Zhao S., Zhou J., Yan J., Wang L., Du X., Li H., Chen Y., Cai W., Wu J. (2018). Curcumin alleviates DSS-induced colitis via inhibiting NLRP3 inflammsome activation and IL-1β production. Mol. Immunol..

[72] Sun Y., Zhao Y., Yao J., Zhao L., Wu Z., Wang Y., Pan D., Miao H., Guo Q., Lu N. (2015). Wogonoside protects against dextran sulfate sodium-induced experimental colitis in mice by inhibiting NF-κB and NLRP3 inflammasome activation. Biochem. Pharmacol..

[73] Pu Z., Han C., Zhang W., Xu M., Wu Z., Liu Y., Wu M., Sun H., Xie H. (2019). Systematic understanding of the mechanism and effects of Arctigenin attenuates inflammation in dextran sulfate sodium-induced acute colitis through suppression of NLRP3 inflammasome by SIRT1. Am J Transl Res.

[74] Mai C.-T., Wu M.-M., Wang C.-L., Su Z.-R., Cheng Y., Song H. (2018). Palmatine attenuated dextran sulfate sodium (DSS)-induced colitis via promoting mitophagy-mediated NLRP3 inflammasome inactivation. Mol. Immunol..

[75] Wu D., Wu K., Zhu Q., Xiao W., Shan Q., Yan Z., Wu J., Deng B., Xue Y., Gong W. (2018). Formononetin Administration Ameliorates Dextran Sulfate Sodium-Induced Acute Colitis by Inhibiting NLRP3 Inflammasome Signaling Pathway. Mediat. Inflamm..

[76] Qu S., Shen Y., Wang M., Wang X., Yang Y. (2019). Suppression of miR-21 and miR-155 of macrophage by cinnamaldehyde ameliorates ulcerative colitis. Int. Immunopharmacol..

[77] Mao L., Kitani A., Strober W., Fuss I.J. (2018). The Role of NLRP3 and IL-1β in the Pathogenesis of Inflammatory Bowel Disease. Front. Immunol..

[78] Huang M., Wang Q., Long F., Di Y., Wang J., Zhu Y.Z., Liu X. (2020). Jmjd3 regulates inflammasome activation and aggravates DSS-induced colitis in mice. FASEB J..

[79] Villani A.-C., Lemire M., Fortin G., Louis E., Silverberg M.S., Collette C., Baba N., Libioulle C., Belaiche J., Bitton A. (2008). Common variants in the NLRP3 region contribute to Crohn’s disease susceptibility. Nat. Genet..

[80] Cocco M., Pellegrini C., Martínez-Banaclocha H., Giorgis M., Marini E., Costale A., Miglio G., Fornai M., Antonioli L., Lopez-Castejon G. (2017). Development of an Acrylate Derivative Targeting the NLRP3 Inflammasome for the Treatment of Inflammatory Bowel Disease. J. Med. Chem..

[81] Pellegrini C., Fornai M., Colucci R.L., Benvenuti L., D’Antongiovanni V., Natale G., Fulceri F., Giorgis M., Marini E., Gastaldi S. (2018). A Comparative Study on the Efficacy of NLRP3 Inflammasome Signaling Inhibitors in a Pre-clinical Model of Bowel Inflammation. Front. Pharmacol..

[82] Zhen Y., Zhang H. (2019). NLRP3 Inflammasome and Inflammatory Bowel Disease. Front. Immunol..

[83] Moossavi M., Parsamanesh N., Bahrami A., Atkin S.L., Sahebkar A. (2018). Role of the NLRP3 inflammasome in cancer. Mol. Cancer.

[84] Karki R., Man S.M., Kanneganti T.-D. (2017). Inflammasomes and Cancer. Cancer Immunol. Res..

[85] Wang B., Li H., Wang X., Zhu X. (2020). The association of aberrant expression of NLRP3 and p-S6K1 in colorectal cancer. Pathol. - Res. Pr..

[86] Ungerbäck J., Belenki D., Ul-Hassan A.J., Fredrikson M., Fransén K., Elander N., Verma D., Söderkvist P. (2012). Genetic variation and alterations of genes involved in NFκB/TNFAIP3- and NLRP3-inflammasome signaling affect susceptibility and outcome of colorectal cancer. Carcinog..

[87] Wang H., Wang Y., Du Q., Lu P., Fan H., Lu J., Hu R. (2016). Inflammasome-independent NLRP3 is required for epithelial-mesenchymal transition in colon cancer cells. Exp. Cell Res..

[88] Shinde R., McGaha T.L. (2018). The Aryl Hydrocarbon Receptor: Connecting Immunity to the Microenvironment. Trends Immunol..

[89] Lamas B., Natividad J.M., Sokol H. (2018). Aryl hydrocarbon receptor and intestinal immunity. Mucosal Immunol..

[90] Denison M.S., Nagy S.R. (2003). Activation of the Aryl Hydrocarbon Receptor by Structurally Diverse Exogenous and Endogenous Chemicals. Annu. Rev. Pharmacol. Toxicol..

[91] Nguyen L.P., Bradfield C.A. (2008). The Search for Endogenous Activators of the Aryl Hydrocarbon Receptor. Chem. Res. Toxicol..

[92] Murray I.A., Perdew G. (2017). Ligand activation of the Ah receptor contributes to gastrointestinal homeostasis. Curr. Opin. Toxicol..

[93] Gutiérrez-Vázquez C., Quintana F.J. (2018). Regulation of the Immune Response by the Aryl Hydrocarbon Receptor. Immun..

[94] Stockinger B., Di Meglio P., Gialitakis M., Duarte J.H. (2014). The Aryl Hydrocarbon Receptor: Multitasking in the Immune System. Annu. Rev. Immunol..

[95] Zhou L. (2016). AHR Function in Lymphocytes: Emerging Concepts. Trends Immunol..

[96] Smirnova A., Wincent E., Bergander L.V., Alsberg T., Bergman J., Rannug A., Rannug U., Vincent E.E. (2015). Evidence for New Light-Independent Pathways for Generation of the Endogenous Aryl Hydrocarbon Receptor Agonist FICZ. Chem. Res. Toxicol..

[97] Bock K.W. (2017). From dioxin toxicity to putative physiologic functions of the human Ah receptor in homeostasis of stem/progenitor cells. Biochem. Pharmacol..

[98] Bock K.W. (2018). From TCDD-mediated toxicity to searches of physiologic AHR functions. Biochem. Pharmacol..

[99] Zelante T., Iannitti R.G., Cunha C., De Luca A., Giovannini G., Pieraccini G., Zecchi R., D’Angelo C., Massi-Benedetti C., Fallarino F. (2013). Tryptophan Catabolites from Microbiota Engage Aryl Hydrocarbon Receptor and Balance Mucosal Reactivity via Interleukin-22. Immun..

[100] Schroeder J.C., DiNatale B.C., Murray I.A., Flaveny C.A., Liu Q., Laurenzana E.M., Lin J.M., Strom S., Omiecinski C.J., Amin S. (2010). The Uremic Toxin 3-Indoxyl Sulfate Is a Potent Endogenous Agonist for the Human Aryl Hydrocarbon Receptor. Biochem..

[101] Jin U.-H., Lee S.-O., Sridharan G., Lee K., Davidson L.A., Jayaraman A., Chapkin R.S., Alaniz R., Safe S. (2014). Microbiome-derived tryptophan metabolites and their aryl hydrocarbon receptor-dependent agonist and antagonist activities. Mol. Pharmacol..

[102] Sinal C.J., Bend J.R. (1997). Aryl Hydrocarbon Receptor-Dependent Induction of Cyp1a1 by Bilirubin in Mouse Hepatoma Hepa 1c1c7 Cells. Mol. Pharmacol..

[103] Phelan D., Winter G., Rogers W., Lam J., Denison M. (1998). Activation of the Ah Receptor Signal Transduction Pathway by Bilirubin and Biliverdin. Arch. Biochem. Biophys..

[104] Kroetz D.L., Zeldin D. (2002). Cytochrome P450 pathways of arachidonic acid metabolism. Curr. Opin. Lipidol..

[105] Seeds M.C., Bass D.A. (1999). Regulation and metabolism of arachidonic acid. Clin. Rev. Allergy Immunol..

[106] Mufti N., Shuler M. (1996). Possible Role of Arachidonic Acid in Stress-Induced Cytochrome P450IA1 Activity. Biotechnol. Prog..

[107] Puga A., Hoffer A., Zhou S., Bohm J.M., Leikauf G., Shertzer H.G. (1997). Sustained Increase in Intracellular Free Calcium and Activation of Cyclooxygenase-2 Expression in Mouse Hepatoma Cells Treated with Dioxin. Biochem. Pharmacol..

[108] Seidel S.D., Winters G.M., Rogers W.J., Ziccardi M.H., Li V., Keser B., Denison M.S. (2001). Activation of the Ah receptor signaling pathway by prostaglandins. J. Biochem. Mol. Toxicol..

[109] Schaldach C.M., Riby J., Bjeldanes L.F. (1999). Lipoxin A4: A New Class of Ligand for the Ah Receptor. Biochem..

[110] Hao N., Whitelaw M.L. (2013). The emerging roles of AhR in physiology and immunity. Biochem. Pharmacol..

[111] Enan E., Matsumura F. (1996). Identification of c-Src as the integral component of the cytosolic Ah receptor complex, transducing the signal of 2,3,7,8-tetrachlorodibenzo-p-dioxin (TCDD) through the protein phosphorylation pathway. Biochem. Pharmacol..

[112] Park S., Dong B., Matsumura F. (2007). Rapid Activation of c-Src Kinase by Dioxin Is Mediated by the Cdc37−HSP90 Complex as Part of Ah Receptor Signaling in MCF10A Cells. Biochemistry.

[113] Mazina O., Park S., Sano H., Wong P., Matsumura F. (2005). Studies on the mechanism of rapid activation of protein tyrosine phosphorylation activities, particularly c-Src kinase, by TCDD in MCF10A. J. Biochem. Mol. Toxicol..

[114] Park S., Mazina O., Kitagawa A., Wong P., Matsumura F. (2005). TCDD causes suppression of growth and differentiation of MCF10A, human mammary epithelial cells by interfering with their insulin receptor signaling through c-Src kinase and ERK activation. J. Biochem. Mol. Toxicol..

[115] Soshilov A., Denison M.S. (2011). Ligand Displaces Heat Shock Protein 90 from Overlapping Binding Sites within the Aryl Hydrocarbon Receptor Ligand-binding Domain*. J. Boil. Chem..

[116] Lees M.J., Peet D., Whitelaw M.L. (2003). Defining the Role for XAP2 in Stabilization of the Dioxin Receptor. J. Boil. Chem..

[117] Tsuji N., Fukuda K., Nagata Y., Okada H., Haga A., Hatakeyama S., Yoshida S., Okamoto T., Hosaka M., Sekine K. (2014). The activation mechanism of the aryl hydrocarbon receptor (AhR) by molecular chaperone HSP90. FEBS Open Bio.

[118] Larigot L., Juricek L., Dairou J., Coumoul X. (2018). AhR signaling pathways and regulatory functions. Biochim. Open.

[119] Guerrina N., Traboulsi H., Eidelman D.H., Baglole C.J. (2018). The Aryl Hydrocarbon Receptor and the Maintenance of Lung Health. Int. J. Mol. Sci..

[120] Vogel C.F.A., Sciullo E., Matsumura F. (2007). Involvement of RelB in aryl hydrocarbon receptor-mediated induction of chemokines. Biochem. Biophys. Res. Commun..

[121] Ahmed S., Valen E., Sandelin A., Matthews J. (2009). Dioxin Increases the Interaction Between Aryl Hydrocarbon Receptor and Estrogen Receptor Alpha at Human Promoters. Toxicol. Sci..

[122] Ohtake F., Baba A., Takada I., Okada M., Iwasaki K., Miki H., Takahashi S., Kouzmenko A., Nohara K., Chiba T. (2007). Dioxin receptor is a ligand-dependent E3 ubiquitin ligase. Nat..

[123] Ohtake F., Takeyama K.-I., Matsumoto T., Kitagawa H., Yamamoto Y., Nohara K., Tohyama C., Krust A., Mimura J., Chambon P. (2003). Modulation of oestrogen receptor signalling by association with the activated dioxin receptor. Nat..

[124] Puga A., Ma C., Marlowe J.L. (2008). The aryl hydrocarbon receptor cross-talks with multiple signal transduction pathways. Biochem. Pharmacol..

[125] Puga A., Barnes S.J., Dalton T.P., Chang C.-Y., Knudsen E.S., Maier M.A. (2000). Aromatic hydrocarbon receptor interaction with the retinoblastoma protein potentiates repression of E2F-dependent transcription and cell cycle arrest. J. Boil. Chem..

[126] Marlowe J.L., Knudsen E.S., Schwemberger S., Puga A. (2004). The Aryl Hydrocarbon Receptor Displaces p300 from E2F-dependent Promoters and Represses S Phase-specific Gene Expression. J. Boil. Chem..

[127] Bureau C., Hanoun N., Torrisani J., Vinel J.-P., Buscail L., Cordelier P. (2009). Expression and Function of Kruppel Like-Factors (KLF) in Carcinogenesis. Curr. Genom..

[128] Jackson D.P., Li H., Mitchell K.A., Joshi A.D., Elferink C.J. (2014). Ah receptor-mediated suppression of liver regeneration through NC-XRE-driven p21Cip1 expression. Mol. Pharmacol..

[129] Wright E.J., De Castro K.P., Joshi A.D., Elferink C.J. (2017). Canonical and non-canonical aryl hydrocarbon receptor signaling pathways. Curr. Opin. Toxicol..

[130] Huang G., Elferink C.J. (2011). A novel nonconsensus xenobiotic response element capable of mediating aryl hydrocarbon receptor-dependent gene expression. Mol. Pharmacol..

[131] Davarinos N.A., Pollenz R.S. (1999). Aryl Hydrocarbon Receptor Imported into the Nucleus following Ligand Binding Is Rapidly Degraded via the Cytosplasmic Proteasome following Nuclear Export. J. Boil. Chem..

[132] Vogel C.F.A., Haarmann-Stemmann T. (2017). The aryl hydrocarbon receptor repressor – More than a simple feedback inhibitor of AhR signaling: Clues for its role in inflammation and cancer. Curr. Opin. Toxicol..

[133] MacPherson L., Ahmed S., Tamblyn L., Krutmann J., Förster I., Weighardt H., Matthews J. (2014). Aryl Hydrocarbon Receptor Repressor and TiPARP (ARTD14) Use Similar, but also Distinct Mechanisms to Repress Aryl Hydrocarbon Receptor Signaling. Int. J. Mol. Sci..

[134] MacPherson L., Tamblyn L., Rajendra S., Bralha F., McPherson J.P., Matthews J. (2012). 2,3,7,8-Tetrachlorodibenzo-p-dioxin poly(ADP-ribose) polymerase (TiPARP, ARTD14) is a mono-ADP-ribosyltransferase and repressor of aryl hydrocarbon receptor transactivation. Nucleic Acids Res..

[135] Gomez A., Bindesbøll C., Satheesh S.V., Grimaldi G., Hutin D., MacPherson L., Ahmed S., Tamblyn L., Cho T., Nebb H.I. (2018). Characterization of TCDD-inducible poly-ADP-ribose polymerase (TIPARP/ARTD14) catalytic activity. Biochem. J..

[136] Diani-Moore S., Zhang S., Ram P., Rifkind A.B. (2013). Aryl Hydrocarbon Receptor Activation by Dioxin Targets Phosphoenolpyruvate Carboxykinase (PEPCK) for ADP-ribosylation via 2,3,7,8-Tetrachlorodibenzo-p-dioxin (TCDD)-inducible Poly(ADP-ribose) Polymerase (TiPARP)*. J. Boil. Chem..

[137] Sorg O., Zennegg M., Schmid P., Fedosyuk R., Valikhnovskyi R., Gaide O., Kniazevych V., Saurat J.-H. (2009). 2,3,7,8-tetrachlorodibenzo-p-dioxin (TCDD) poisoning in Victor Yushchenko: Identification and measurement of TCDD metabolites. Lancet.

[138] McKee M. (2009). The poisoning of Victor Yushchenko. Lancet.

[139] Wang Z., Monti S., Sherr D.H. (2017). The diverse and important contributions of the AHR to cancer and cancer immunity. Curr. Opin. Toxicol..

[140] Neavin D., Liu D., Ray B., Weinshilboum R.M. (2018). The Role of the Aryl Hydrocarbon Receptor (AHR) in Immune and Inflammatory Diseases. Int. J. Mol. Sci..

[141] Liu T., Zhang L., Joo D., Sun S. (2017). NF-κB signaling in inflammation. Signal Transduct. Target. Ther..

[142] Qiao Y., Wang P., Qi J., Zhang L., Gao C. (2012). TLR-induced NF-κB activation regulates NLRP3 expression in murine macrophages. FEBS Lett..

[143] Afonina I.S., Zhong Z., Karin M., Beyaert R. (2017). Limiting inflammation—the negative regulation of NF-κB and the NLRP3 inflammasome. Nat. Immunol..

[144] Kimura A., Abe H., Tsuruta S., Chiba S., Fujii-Kuriyama Y., Sekiya T., Morita R., Yoshimura A. (2013). Aryl hydrocarbon receptor protects against bacterial infection by promoting macrophage survival and reactive oxygen species production. Int. Immunol..

[145] Jia Y., Zhang C., Hua M., Wang M., Chen P., Ma D. (2017). Aberrant NLRP3 inflammasome associated with aryl hydrocarbon receptor potentially contributes to the imbalance of T-helper cells in patients with acute myeloid leukemia. Oncol. Lett..

[146] Bauernfeind F., Rieger A., Schildberg F.A., Knolle P.A., Schmid-Burgk J.L., Hornung V. (2012). NLRP3 Inflammasome Activity Is Negatively Controlled by miR-223. J. Immunol..

[147] Haneklaus M., Gerlic M., Kurowska-Stolarska M., Rainey A.A., Pich D., McInnes I.B., Hammerschmidt W., O’Neill L.A., Masters S.L. (2012). Cutting edge: miR-223 and EBV miR-BART15 regulate the NLRP3 inflammasome and IL-1beta production. J. Immunol..

[148] Ogando J., Tardáguila M., Díaz-Alderete A., Usategui A., Miranda-Ramos V., Martínez-Herrera D.J., De La Fuente L., García-León M.J., Moreno M.C., Escudero S. (2016). Notch-regulated miR-223 targets the aryl hydrocarbon receptor pathway and increases cytokine production in macrophages from rheumatoid arthritis patients. Sci. Rep..

[149] Tian Y., Ke S., Denison M.S., Rabson A.B., Gallo M.A. (1999). Ah Receptor and NF-κB Interactions, a Potential Mechanism for Dioxin Toxicity. J. Boil. Chem..

[150] Sheppard K.-A., Phelps K.M., Williams A.J., Thanos D., Glass C.K., Rosenfeld M.G., Gerritsen M.E., Collins T. (1998). Nuclear Integration of Glucocorticoid Receptor and Nuclear Factor- B Signaling by CREB-binding Protein and Steroid Receptor Coactivator-1. J. Boil. Chem..

[151] Ishihara Y., Kado S.Y., Hoeper C., Harel S., Vogel C.F.A. (2019). Role of NF-kB RelB in Aryl Hydrocarbon Receptor-Mediated Ligand Specific Effects. Int. J. Mol. Sci..

[152] Vogel C.F.A., Sciullo E., Li W., Wong P., Lazennec G., Matsumura F. (2007). RelB, a new partner of aryl hydrocarbon receptor-mediated transcription. Mol. Endocrinol..

[153] Wang L., Zhai D.-S., Ruan B.-J., Xu C.-M., Ye Z.-C., Lu H.-Y., Jiang Y.-H., Wang Z.-Y., Xiang A., Yang Y. (2017). Quaking Deficiency Amplifies Inflammation in Experimental Endotoxemia via the Aryl Hydrocarbon Receptor/Signal Transducer and Activator of Transcription 1–NF-κB Pathway. Front. Immunol..

[154] Huai W., Zhao R., Song H., Zhao J., Zhang L., Zhang L., Gao C., Han L., Zhao W. (2014). Aryl hydrocarbon receptor negatively regulates NLRP3 inflammasome activity by inhibiting NLRP3 transcription. Nat. Commun..

[155] Kimura A., Naka T., Nakahama T., Chinen I., Masuda K., Nohara K., Fujii-Kuriyama Y., Kishimoto T. (2009). Aryl hydrocarbon receptor in combination with Stat1 regulates LPS-induced inflammatory responses. J. Exp. Med..

[156] Sekine H., Mimura J., Oshima M., Okawa H., Kanno J., Igarashi K., Gonzalez F.J., Ikuta T., Kawajiri K., Fujii-Kuriyama Y. (2009). Hypersensitivity of Aryl Hydrocarbon Receptor-Deficient Mice to Lipopolysaccharide-Induced Septic Shock. Mol. Cell. Boil..

[157] Zhu J., Luo L., Tian L., Yin S., Ma X., Cheng S., Tang W., Yu J., Ma W., Zhou X. (2018). Aryl Hydrocarbon Receptor Promotes IL-10 Expression in Inflammatory Macrophages Through Src-STAT3 Signaling Pathway. Front. Immunol..

[158] Wakamatsu T., Yamamoto S., Ito T., Sato Y., Matsuo K., Takahashi Y., Kaneko Y., Goto S., Kazama J.J., Gejyo F. (2018). Indoxyl Sulfate Promotes Macrophage IL-1β Production by Activating Aryl Hydrocarbon Receptor/NF-κ/MAPK Cascades, but the NLRP3 inflammasome Was Not Activated. Toxins.

[159] Benson J.M., Shepherd D.M. (2011). Aryl hydrocarbon receptor activation by TCDD reduces inflammation associated with Crohn’s disease. Toxicol. Sci..

[160] Takamura T., Harama D., Fukumoto S., Nakamura Y., Shimokawa N., Ishimaru K., Ikegami S., Makino S., Kitamura M., Nakao A. (2011). Lactobacillus bulgaricus OLL1181 activates the aryl hydrocarbon receptor pathway and inhibits colitis. Immunol. Cell Boil..

[161] Monteleone I., Macdonald T.T., Pallone F., Monteleone G. (2012). The aryl hydrocarbon receptor in inflammatory bowel disease. Curr. Opin. Gastroenterol..

[162] Alzahrani A.M., Hanieh H., Ibrahim H.-I.M., Mohafez O., Shehata T.M., Ismail M.B., Al-Fwuaires M. (2017). Enhancing miR-132 expression by aryl hydrocarbon receptor attenuates tumorigenesis associated with chronic colitis. Int. Immunopharmacol..

[163] Singh N.P., Singh U.P., Singh B., Price R.L., Nagarkatti M., Nagarkatti P.S. (2011). Activation of Aryl Hydrocarbon Receptor (AhR) Leads to Reciprocal Epigenetic Regulation of FoxP3 and IL-17 Expression and Amelioration of Experimental Colitis. PLoS ONE.

[164] Monteleone I., Rizzo A., Sarra M., Sica G., Sileri P., Biancone L., Macdonald T.T., Pallone F., Monteleone G. (2011). Aryl Hydrocarbon Receptor-Induced Signals Up-regulate IL-22 Production and Inhibit Inflammation in the Gastrointestinal Tract. Gastroenterol..

[165] Takamura T., Harama D., Matsuoka S., Shimokawa N., Nakamura Y., Okumura K., Ogawa H., Kitamura M., Nakao A. (2010). Activation of the aryl hydrocarbon receptor pathway may ameliorate dextran sodium sulfate-induced colitis in mice. Immunol. Cell Boil..

[166] Furumatsu K., Nishiumi S., Kawano Y., Ooi M., Yoshie T., Shiomi Y., Kutsumi H., Ashida H., Fujii-Kuriyama Y., Azuma T. (2011). A Role of the Aryl Hydrocarbon Receptor in Attenuation of Colitis. Dig. Dis. Sci..

[167] Arsenescu R., Arsenescu V., Zhong J., Nasser M., Melinte R., Dingle R.W.C., Swanson H., De Villiers W.J. (2010). Role of the xenobiotic receptor in inflammatory bowel disease. Inflamm. Bowel Dis..

[168] Duan C., Guo J.-M., Dai Y., Xia Y.-F. (2017). The absorption enhancement of norisoboldine in the duodenum of adjuvant-induced arthritis rats involves the impairment of P-glycoprotein. Biopharm. Drug Dispos..

[169] Lv Q., Wang K., Qiao S.-M., Dai Y., Wei Z. (2018). Norisoboldine, a natural aryl hydrocarbon receptor agonist, alleviates TNBS-induced colitis in mice, by inhibiting the activation of NLRP3 inflammasome. Chin. J. Nat. Med..

[170] Lv Q., Wang K., Qiao S., Yang L., Xin Y., Dai Y., Wei Z. (2018). Norisoboldine, a natural AhR agonist, promotes Treg differentiation and attenuates colitis via targeting glycolysis and subsequent NAD+/SIRT1/SUV39H1/H3K9me3 signaling pathway. Cell Death Dis..

[171] Tong B., Yuan X., Dou Y., Wu X., Chou G., Wang Z., Xia Y., Dai Y., Daia Y. (2016). Norisoboldine, an isoquinoline alkaloid, acts as an aryl hydrocarbon receptor ligand to induce intestinal Treg cells and thereby attenuate arthritis. Int. J. Biochem. Cell Boil..

[172] Liu X., Zhang X., Ding Y., Zhou W., Tao L., Lu P., Wang Y., Hu R. (2016). Nuclear Factor E2-Related Factor-2 Negatively Regulates NLRP3 Inflammasome Activity by Inhibiting Reactive Oxygen Species-Induced NLRP3 Priming. Antioxidants Redox Signal..

[173] Wang Z., Tang X., Wu X., Yang M., Wang W., Wang L., Tang N., Wang D. (2019). Cardamonin exerts anti-gastric cancer activity via inhibiting LncRNA-PVT1-STAT3 axis. Biosci. Rep..

[174] Wang Z., Xu G., Gao Y., Zhan X., Qin N., Fu S., Li R., Niu M., Wang J., Liu Y. (2019). Cardamonin from a medicinal herb protects against LPS-induced septic shock by suppressing NLRP3 inflammasome. Acta Pharm. Sin. B.

[175] Wang K., Lv Q., Miao Y.-M., Qiao S.-M., Dai Y., Wei Z. (2018). Cardamonin, a natural flavone, alleviates inflammatory bowel disease by the inhibition of NLRP3 inflammasome activation via an AhR/Nrf2/NQO1 pathway. Biochem. Pharmacol..

[176] Harper P.A., Prokipcak R.D., Bush L.E., Golas C.L., Okey A.B. (1991). Detection and characterization of the Ah receptor for 2,3,7,8-tetrachlorodibenzo-p-dioxin in the human colon adenocarcinoma cell line LS180. Arch. Biochem. Biophys..

[177] Erdelyi I., Levenkova N., Lin E.Y., Pinto J.T., Lipkin M., Quimby F.W., Holt P. (2009). Western-style diets induce oxidative stress and dysregulate immune responses in the colon in a mouse model of sporadic colon cancer. J. Nutr..

[178] Díaz-Díaz C.J., Ronnekleiv-Kelly S., Nukaya M., Geiger P.G., Balbo S., Dator R., Megna B., Carney P., Bradfield C.A., Kennedy G.D. (2016). The Aryl Hydrocarbon Receptor is a Repressor of Inflammation-associated Colorectal Tumorigenesis in Mouse. Ann. Surg..

[179] Ikuta T., Kobayashi Y., Kitazawa M., Shiizaki K., Itano N., Noda T., Pettersson S., Poellinger L., Fujii-Kuriyama Y., Taniguchi S. (2013). ASC-associated inflammation promotes cecal tumorigenesis in aryl hydrocarbon receptor-deficient mice. Carcinog..

[180] Huang C.-R., Lee C.-T., Chang K.-Y., Chang W.-C., Liu Y.-W., Lee J.-C., Chen B.-K. (2015). Down-regulation of ARNT promotes cancer metastasis by activating the fibronectin/integrin β1/FAK axis. Oncotarget.

